# Supplementary material for: FABP7 Facilitates Uptake of Docosahexaenoic Acid in Glioblastoma Neural Stem-like Cells
Source: Nutrients. 2021 Jul 30;13(8):2664. doi: 10.3390/nu13082664 (PMC8402214; doi:10.3390/nu13082664)
Supplement: Supplementary file 1 [file nutrients-13-02664-s001.zip › nutrients-1302449-supplementary.pdf]

Supplementary Materials

FABP7 Facilitates Uptake of Docosahexaenoic Acid in Glioblastoma Neural Stem-like Cells

Won-Shik Choi[1*], Xia Xu[1*], Susan Goruk[2], Yixiong Wang[1], Samir Patel[1], Michael Chow[3], Catherine J. Field[2] and Roseline Godbout[1#]

[1]Department of Oncology, Cross Cancer Institute, University of Alberta, 11560 University Avenue, Edmonton, AB T6G 1Z2, Canada; [2]Department of Agricultural, Food and Nutritional Science, University of Alberta, Edmonton, AB T6G 2E1, Canada; and [3]Department of Surgery, University of Alberta, Edmonton, AB T6G 2B7, Canada

* These authors made equal contributions to the manuscript
# Communicating author

Table of contents

Figure S1: Effects of DHA treatment on lipid droplet formation in U251 and ED501N cells with or without FABP7 depletion.

Table S1: Fatty acid composition of total lipids extracted from A4-004N and A4-004Adh cells.

Table S2: Fatty acid composition of total lipids extracted from A4-004N and A4-004Adh cells cultured in media supplemented with BSA (control), 30 µM AA or 30 µM DHA.

Table S3: Fatty acid composition of total lipids and total phospholipids extracted from A4-004N and A4-004Adh cells cultured under normal culture conditions and in media supplemented with 30 µM BSA.

Table S4: Fatty acid composition of total phospholipids from A4-004N and A4-004Adh cells.

Table S5: Fatty acid composition of total phospholipids from A4-004N and A4-004Adh cells cultured in media supplemented with BSA (control), 30 µM AA or 30 µM DHA.

Figure S1

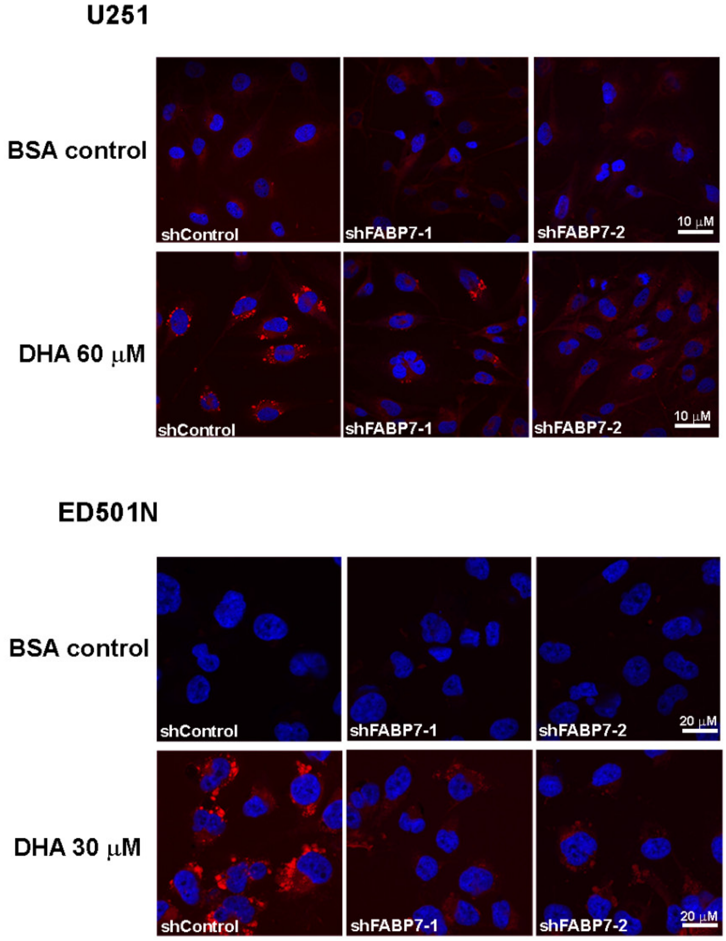

Supplementary Figure S1. Effect of DHA treatment and FABP7-knockdown on lipid droplet formation in U251 and ED501N cells. Stable knockdown of FABP7 in U251 GBM adherent cells and ED501 GBM neural stem-like cells was confirmed as previously described [49]. Cells were cultured in medium supplemented with BSA (control), 60 μM DHA (U251 cells), or 30 μM DHA (ED501N cells). After 24 hours, cells were fixed and stained with Nile Red. Representative confocal images are shown. DAPI was used to stain the nucleus.

Table S1. Fatty acid composition of total lipids extracted from A4-004N and A4-004Adh cells.

Fatty Acids	A4-004N (N = 3)		A4-004Adh (N = 3)		
	% Abundance of fatty acids in total lipids				
	Mean	±SD	Mean	±SD	*p*-value
C14:0	2.14	±0.10	1.71	±0.07	*p = 0.53*
C15:0	0.13	±0.02	0.52	±0.03	*p = 0.0001*
C16:0 (PA)	25.75	±0.59	29.22	±1.08	*p = 0.008*
C17:0	0.89	±0.06	0.67	±0.06	*p = 0.01*
C18:0 (SA)	15.74	±0.11	17.87	±1.15	*p = 0.03*
C20:0	0.21	±0.04	0.39	±0.07	*p = 0.01*
C24:0	4.37	±0.40	0.63	±0.13	*p = 0.0001*
Total SFA	49.24	±0.27	52.39	±1.48	*p = 0.02*
C16:1ω-9	7.88	±0.16	6.14	±0.50	*p = 0.004*
C18:1ω-9 (OA)	27.45	±0.48	21.94	±0.66	*p = 0.0003*
C18:1ω-7	5.65	±0.09	7.37	±0.25	*p = 0.0004*
C24:1ω-9	1.42	±0.51	1.15	±0.17	*p = 0.44*
Total MUFA	42.40	±0.09	36.59	±1.26	*p = 0.001*
C18:2ω-6 (LA)	0.18	±0.03	0.72	±0.09	*p = 0.0005*
C20:2ω-6	3.25	±0.24	0.95	±0.14	*p = 0.0001*
C20:3ω-6 (DGLA)	0.74	±0.10	1.67	±0.07	*p = 0.0002*
C20:4ω-6 (AA)	1.01	±0.14	3.01	±0.36	*p = 0.0008*
C22:4ω-6 (ADA)	0.60	±0.01	1.24	±0.13	*p = 0.001*
C22:5ω-6 (ω-6 DPA)	0.10	±0.03	0.22	±0.03	*p = 0.006*
Total ω-6	5.88	±0.29	7.82	±0.47	*p = 0.004*
C18:3ω-3 (ALA)	1.79	±0.09	0.95	±0.08	*p = 0.0003*
C20:4ω-3	0.06	±0.02	0.16	±0.01	*p = 0.001*
C20:5ω-3 (EPA)	0.09	±0.03	0.09	±0.02	*p = 0.88*
C22:5ω-3 (ω-3 DPA)	0.47	±0.04	0.96	±0.11	*p = 0.002*
C22:6ω-3 (DHA)	0.09	±0.01	1.04	±0.13	*p = 0.0002*
Total ω-3	2.49	±0.05	3.20	±0.19	*p = 0.003*
Total PUFA	8.36	±0.34	11.02	±0.65	*p = 0.003*
C22:6ω-3:C20:4ω-6	0.14	±0.02	0.84	±0.05	*p < 0.0001*
ω-3 PUFA:ω-6 PUFA	0.42	±0.01	0.41	±0.00	*p = 0.13*

Data are presented as mean ± standard deviation (SD). *p*-value of <0.05 was considered statistically significant.

Table S2. Fatty acid composition of total lipids extracted from A4-004N and A4-004Adh cells cultured in media supplemented with BSA (control), 30 μM AA or 30 μM DHA.

Fatty Acids	A4-004N							A4-004Adh						
	BSA Control (N = 3)		AA 30 μM (N = 3)		DHA 30 μM (N = 3)			BSA Control (N = 3)		AA 30 μM (N = 3)		DHA 30 μM (N = 3)		
	% Abundance of fatty acids in total lipids													
	Average	±SD	Average	±SD	Average	±SD	ANOVA	Average	±SD	Average	±SD	Average	±SD	ANOVA
C14:0	2.21a	±0.08	2.65b	±0.05	2.18a	±0.03	$p = 0.0001$	1.89a	±0.02	1.73ab	±0.07	1.68b	±0.11	$p = 0.036$
C15:0	0.16	±0.02	0.14	±0.02	0.12	±0.03	$p = 0.14$	0.55a	±0.01	0.48b	±0.02	0.50b	±0.02	$p = 0.0066$
C16:0 (PA)	25.54a	±0.24	25.53a	±0.40	24.23b	±0.42	$p = 0.0065$	29.38a	±0.46	30.52b	±0.36	29.61ab	±0.41	$p = 0.033$
C17:0	0.81a	±0.06	0.83a	±0.04	0.55b	±0.07	$p = 0.0012$	0.60	±0.12	0.64	±0.04	0.70	±0.04	$p = 0.38$
C18:0 (SA)	16.39a	±0.46	17.74b	±0.48	17.47ab	±0.54	$p = 0.034$	17.30a	±0.40	18.45b	±0.07	17.91ab	±0.29	$p = 0.027$
C20:0	0.29	±0.05	0.24	±0.03	0.24	±0.03	$p = 0.17$	0.36a	±0.03	0.29b	±0.02	0.29b	±0.01	$p = 0.014$
C24:0	3.91a	±0.44	2.52b	±0.29	2.72b	±0.06	$p = 0.0082$	0.77a	±0.03	0.34b	±0.06	0.39b	±0.06	$p = 0.0001$
Total SFA	49.30	±0.71	49.65	±0.45	47.79	±1.27	$p = 0.10$	50.85	±0.98	52.22	±0.22	51.08	±0.73	$p = 0.23$
C16:1ω-9	7.43a	±0.14	5.75b	±0.05	5.46b	±0.49	$p = 0.0004$	5.76a	±0.25	3.74b	±0.19	4.10b	±0.23	$p < 0.0001$
C18:1ω-9 (OA)	27.27a	±0.37	20.67b	±0.24	23.39c	±0.52	$p < 0.0001$	22.31a	±0.47	15.64b	±0.54	18.70c	±0.56	$p < 0.0001$
C18:1ω-7	5.85a	±0.03	4.33b	±0.13	3.98b	±0.41	$p = 0.0002$	7.25a	±0.16	5.81b	±0.13	5.76b	±0.19	$p < 0.0001$
C24:1ω-9	1.25	±0.16	0.98	±0.10	0.95	±0.13	$p = 0.058$	1.47a	±0.05	0.78b	±0.22	1.05b	±0.02	$p = 0.0018$
Total MUFA	41.80a	±0.62	31.73b	±0.46	34.22c	±0.73	$p < 0.0001$	36.79a	±0.90	25.97b	±0.98	29.60c	±0.95	$p < 0.0001$
C18:2ω-6 (LA)	0.34a	±0.01	0.16b	±0.03	0.79c	±0.20	$p < 0.0001$	1.20a	±0.14	0.68b	±0.07	1.81c	±0.08	$p < 0.0001$
C20:2ω-6	2.72a	±0.22	1.75b	±0.20	1.83b	±0.28	$p = 0.0042$	0.82a	±0.15	0.35b	±0.05	0.39b	±0.05	$p = 0.0017$
C20:3ω-6 (DGLA)	0.94	±0.09	1.06	±0.07	1.07	±0.11	$p = 0.22$	2.20a	±0.11	1.54b	±0.05	2.05a	±0.09	$p = 0.0002$
C20:4ω-6 (AA)	1.47a	±0.04	6.31b	±0.19	1.18a	±0.09	$p < 0.0001$	3.43a	±0.09	7.87b	±0.75	3.01a	±0.16	$p < 0.0001$
C22:4ω-6 (ADA)	0.87a	±0.06	6.77b	±0.12	0.58c	±0.07	$p < 0.0001$	1.40a	±0.03	6.81b	±0.79	1.12a	±0.08	$p < 0.0001$
C22:5ω-6 (ω-6 DPA)	0.10a	±0.01	0.76b	±0.07	0.11a	±0.03	$p < 0.0001$	0.19a	±0.01	1.04b	±0.09	0.24a	±0.04	$p < 0.0001$
Total ω-6	6.45a	±0.29	16.80b	±0.07	5.66c	±0.09	$p < 0.0001$	9.24a	±0.35	18.30b	±1.55	8.63a	±0.20	$p < 0.0001$
C18:3ω-3 (ALA)	1.68a	±0.04	1.19b	±0.06	1.13b	±0.12	$p = 0.0003$	0.91a	±0.04	0.60b	±0.08	0.63b	±0.03	$p = 0.0006$
C20:4ω-3	0.08	±0.02	0.05	±0.01	0.04	±0.01	$p = 0.12$	0.19a	±0.02	0.11b	±0.03	0.10b	±0.02	$p = 0.014$
C20:5ω-3 (EPA)	0.07a	±0.01	0.07a	±0.01	0.41b	±0.08	$p = 0.0001$	0.09a	±0.01	0.07a	±0.01	0.45b	±0.03	$p < 0.0001$
C22:5ω-3 (ω-3 DPA)	0.52a	±0.06	0.42a	±0.03	1.21b	±0.18	$p = 0.0003$	0.92a	±0.05	0.86a	±0.10	1.42b	±0.09	$p = 0.0003$
C22:6ω-3 (DHA)	0.10a	±0.02	0.07a	±0.01	11.20b	±2.76	$p = 0.0002$	1.01a	±0.09	0.90a	±0.11	8.09b	±0.29	$p < 0.0001$
Total ω-3	2.45a	±0.06	1.81a	±0.06	13.99b	±2.91	$p = 0.0002$	3.12a	±0.07	2.54a	±0.15	10.69b	±0.41	$p < 0.0001$
Total PUFA	8.90a	±0.35	18.62b	±0.01	18.00b	±0.54	$p < 0.0001$	12.36a	±0.37	20.84b	±1.69	19.31b	±0.58	$p = 0.0001$
C22:6ω-3:C20:4ω-6	0.07a	±0.01	0.01a	±0.00	9.65b	±3.13	$p = 0.0009$	0.30a	±0.02	0.11b	±0.01	2.69c	±0.12	$p < 0.0001$
ω-3 PUFA:ω-6 PUFA	0.38a	±0.01	0.11b	±0.00	2.18c	±0.14	$p < 0.0001$	0.34a	±0.01	0.14b	±0.00	1.24c	±0.03	$p < 0.0001$

Different letters indicate that groups are significantly different. Differences were assessed for significance using one-way analysis of variance followed by post-hoc Tukey's test. *p*-value of <0.05 was considered statistically significant.

Table S3. Fatty acid composition of total lipids and total phospholipids from A4-004N and A4-004Adh cells cultured under normal culture conditions (not treated (NT); from Tables S1 and S4) and in media supplemented with 30 µM BSA (from Tables S2 and S5).

Fatty Acids	A4-004N total lipid					A4-004Adh total lipid					A4-004N total phospholipid					A4-004Adh total phospholipid				
	NT		BSA			NT		BSA			NT		BSA			NT		BSA		
	Mean	±SD	Average	±SD	t-test	Mean	±SD	Average	±SD	t-test	Mean	±SD	Average	±SD	t-test	Mean	±SD	Average	±SD	t-test
C14:0	2.14	±0.10	2.21	±0.08	*0.39*	1.71	±0.07	1.89	±0.02	*0.43*	1.92	±0.15	1.56	±0.56	*0.34*	1.47	±0.10	1.48	±0.17	*0.92*
C15:0	0.13	±0.02	0.16	±0.02	*0.21*	0.52	±0.03	0.55	±0.01	*0.35*	0.21	±0.14	0.20	±0.09	*0.93*	0.47	±0.04	0.46	±0.07	*0.88*
C16:0 (PA)	25.75	±0.59	25.54	±0.24	*0.59*	29.22	±1.08	29.38	±0.46	*0.83*	24.59	±0.04	24.50	±0.54	*0.84*	26.23	±0.88	26.18	±0.97	*0.95*
C17:0	0.89	±0.06	0.81	±0.06	*0.17*	0.67	±0.06	0.60	±0.12	*0.43*	0.72	±0.11	0.70	±0.19	*0.89*	0.58	±0.06	0.57	±0.08	*0.91*
C18:0 (SA)	15.74	±0.11	16.39	±0.46	*0.08*	17.87	±1.15	17.30	±0.40	*0.47*	14.86	±0.58	14.53	±0.54	*0.62*	16.40	±0.55	16.24	±0.85	*0.81*
C20:0	0.21	±0.04	0.29	±0.05	*0.08*	0.39	±0.07	0.36	±0.03	*0.55*	0.50	±0.05	0.56	±0.14	*0.55*	0.43	±0.11	0.55	±0.11	*0.32*
C24:0	4.37	±0.40	3.91	±0.44	*0.26*	0.63	±0.13	0.77	±0.03	*0.14*	4.43	±0.15	3.92	±0.57	*0.35*	0.82	±0.22	0.78	±0.15	*0.85*
Total SFA	49.24	±0.27	49.30	±0.71	*0.89*	52.39	±1.48	50.85	±0.98	*0.21*	47.23	±0.13	46.17	±0.21	*0.03*	47.20	±0.10	46.27	±2.10	*0.59*
C16:1ω-9	7.88	±0.16	7.43	±0.14	*0.02*	6.14	±0.50	5.76	±0.25	*0.31*	8.22	±0.15	8.00	±0.06	*0.17*	7.46	±0.39	7.33	±0.38	*0.73*
C18:1ω-9 (OA)	27.45	±0.48	27.27	±0.37	*0.64*	21.94	±0.66	22.31	±0.47	*0.48*	27.29	±0.22	28.14	±0.36	*0.10*	23.66	±0.41	23.56	±0.46	*0.82*
C18:1ω-7	5.65	±0.09	5.85	±0.03	*0.02*	7.37	±0.25	7.25	±0.16	*0.53*	6.18	±0.10	6.26	±0.20	*0.59*	6.96	±0.19	6.93	±0.40	*0.92*
C24:1ω-9	1.42	±0.51	1.25	±0.16	*0.62*	1.15	±0.17	1.47	±0.05	*0.04*	1.35	±0.29	1.40	±0.04	*0.78*	1.11	±0.33	1.23	±0.17	*0.69*
Total MUFA	42.40	±0.09	41.80	±0.62	*0.18*	36.59	±1.26	36.79	±0.90	*0.84*	42.85	±0.22	43.88	±0.20	*0.04*	39.18	±0.92	39.05	±1.41	*0.90*
C18:2ω-6 (LA)	0.18	±0.03	0.34	±0.01	*0.00*	0.72	±0.09	1.20	±0.14	*0.01*	0.39	±0.30	0.33	±0.08	*0.81*	0.97	±0.08	1.00	±0.26	*0.84*
C20:2ω-6	3.25	±0.24	2.72	±0.22	*0.05*	0.95	±0.14	0.82	±0.15	*0.32*	3.91	±0.28	3.99	±0.09	*0.77*	1.27	±0.21	1.27	±0.27	*0.99*
C20:3ω-6 (DGLA)	0.74	±0.10	0.94	±0.09	*0.06*	1.67	±0.07	2.20	±0.11	*0.00*	1.21	±0.37	1.02	±0.07	*0.55*	2.00	±0.06	1.97	±0.20	*0.81*
C20:4ω-6 (AA)	1.01	±0.14	1.47	±0.04	*0.01*	3.01	±0.36	3.43	±0.09	*0.11*	1.14	±0.07	1.01	±0.02	*0.13*	3.80	±0.12	3.96	±0.18	*0.33*
C22:4ω-6 (ADA)	0.60	±0.01	0.87	±0.06	*0.00*	1.24	±0.13	1.40	±0.03	*0.10*	0.56	±0.02	0.49	±0.02	*0.07*	1.36	±0.05	1.42	±0.14	*0.51*
C22:5ω-6 (ω-6 DPA)	0.10	±0.03	0.10	±0.01	*0.84*	0.22	±0.03	0.19	±0.01	*0.12*	0.24	±0.04	0.34	±0.23	*0.62*	0.45	±0.08	0.54	±0.29	*0.61*
Total ω-6	5.88	±0.29	6.45	±0.29	*0.07*	7.82	±0.47	9.24	±0.35	*0.01*	7.07	±0.10	7.04	±0.21	*0.88*	9.86	±0.36	10.18	±0.24	*0.37*
C18:3ω-3 (ALA)	1.79	±0.09	1.68	±0.04	*0.12*	0.95	±0.08	0.91	±0.04	*0.45*	1.65	±0.27	2.01	±0.11	*0.10*	1.15	±0.10	0.98	±0.21	*0.28*
C20:4ω-3	0.06	±0.02	0.08	±0.02	*0.36*	0.16	±0.01	0.19	±0.02	*0.13*	0.16	±0.07	0.19	±0.09	*0.37*	0.36	±0.07	0.38	±0.09	*0.72*
C20:5ω-3 (EPA)	0.09	±0.03	0.07	±0.01	*0.54*	0.09	±0.02	0.09	±0.01	*0.73*	0.20	±0.08	0.14	±0.00	*0.18*	0.22	±0.02	0.26	±0.14	*0.61*
C22:5ω-3 (ω-3 DPA)	0.47	±0.04	0.52	±0.06	*0.23*	0.96	±0.11	0.92	±0.05	*0.62*	0.51	±0.01	0.34	±0.02	*0.01*	1.04	±0.08	1.08	±0.14	*0.69*
C22:6ω-3 (DHA)	0.09	±0.01	0.10	±0.02	*0.40*	1.04	±0.13	1.01	±0.09	*0.78*	0.23	±0.07	0.33	±0.18	*0.72*	1.78	±0.14	1.81	±0.15	*0.85*
Total ω-3	2.49	±0.05	2.45	±0.06	*0.45*	3.20	±0.19	3.12	±0.07	*0.52*	2.86	±0.01	2.85	±0.10	*0.95*	4.55	±0.17	4.51	±0.45	*0.90*
Total PUFA	8.36	±0.34	8.90	±0.35	*0.13*	11.02	±0.65	12.36	±0.37	*0.04*	9.93	±0.09	9.84	±0.25	*0.68*	14.41	±0.47	14.69	±0.69	*0.62*
C22:6ω-3:C20:4ω-6	0.14	±0.02	0.07	±0.01	*0.01*	0.84	±0.05	0.30	±0.02	*0.00*	0.24	±0.04	0.23	±0.09	*0.84*	0.47	±0.02	0.46	±0.02	*0.60*
ω-3 PUFA:ω-6 PUFA	0.42	±0.01	0.38	±0.01	*0.01*	0.41	±0.00	0.34	±0.01	*0.00*	0.40	±0.01	0.40	±0.01	*0.41*	0.46	±0.02	0.44	±0.03	*0.46*

Data are presented as mean ± standard deviation (SD). *p*-value of <0.05 was considered statistically significant. N = 3.

Table S4. **Fatty acid composition of total phospholipids from A4-004N and A4-004Adh cells.**

Fatty Acids	A4-004N (N = 3)		A4-004Adh (N = 3)		
	% Abundance of fatty acids in total phospholipids				
	Mean	±SD	Mean	±SD	*p*-value
C14:0	1.92	±0.15	1.47	±0.10	*p = 0.011*
C15:0	0.21	±0.14	0.47	±0.04	*p = 0.038*
C16:0 (PA)	24.59	±0.04	26.23	±0.88	*p = 0.09*
C17:0	0.72	±0.11	0.58	±0.06	*p = 0.12*
C18:0 (SA)	14.86	±0.58	16.40	±0.55	*p = 0.057*
C20:0	0.50	±0.05	0.43	±0.11	*p = 0.38*
C24:0	4.43	±0.15	0.82	±0.22	*p = 0.0003*
Total SFA	47.23	±0.13	47.20	±0.10	*p = 0.84*
C16:1ω-9	8.22	±0.15	7.46	±0.39	*p = 0.08*
C18:1ω-9 (OA)	27.29	±0.22	23.66	±0.41	*p = 0.0015*
C18:1ω-7	6.18	±0.10	6.96	±0.19	*p = 0.0035*
C24:1ω-9	1.35	±0.29	1.11	±0.33	*p = 0.40*
Total MUFA	42.85	±0.22	39.18	±0.92	*p = 0.013*
C18:2ω-6 (LA)	0.39	±0.30	0.97	±0.08	*p = 0.032*
C20:2ω-6	3.91	±0.28	1.27	±0.21	*p = 0.0012*
C20:3ω-6 (DGLA)	1.21	±0.37	2.00	±0.06	*p = 0.021*
C20:4ω-6 (AA)	1.14	±0.07	3.80	±0.12	*p = 0.0001*
C22:4ω-6 (ADA)	0.56	±0.02	1.36	±0.05	*p = 0.0002*
C22:5ω-6 (ω-6 DPA)	0.24	±0.04	0.45	±0.08	*p = 0.049*
Total ω-6	7.07	±0.10	9.86	±0.36	*p = 0.002*
C18:3ω-3 (ALA)	1.65	±0.27	1.15	±0.10	*p = 0.041*
C20:4ω-3	0.16	±0.07	0.36	±0.07	*p = 0.020*
C20:5ω-3 (EPA)	0.20	±0.08	0.22	±0.02	*p = 0.028*
C22:5ω-3 (ω-3 DPA)	0.51	±0.01	1.04	±0.08	*p = 0.003*
C22:6ω-3 (DHA)	0.23	±0.07	1.78	±0.14	*p = 0.0008*
Total ω-3	2.86	±0.01	4.55	±0.17	*p = 0.0009*
Total PUFA	9.93	±0.09	14.41	±0.47	*p = 0.0010*
C22:6ω-3:C20:4ω-6	0.24	±0.04	0.47	±0.02	*p = 0.0035*
ω-3 PUFA:ω-6 PUFA	0.40	±0.01	0.46	±0.02	*p = 0.025*

Data are presented as mean ± standard deviation (SD). *p*-value of <0.05 was considered statistically significant.

Table S5. Fatty acid composition of total phospholipids from A4-004N and A4-004Adh cells cultured in media supplemented with BSA (control), 30 µM AA or 30 µM DHA.

Fatty Acids	A4-004N							A4-004Adh						
	BSA Control (N = 3)		AA 30 µM (N = 3)		DHA 30 µM (N = 3)			BSA Control (N = 3)		AA 30 µM (N = 3)		DHA 30 µM (N = 3)		
	% Abundance of fatty acids in total phospholipids													
	Average	±SD	Average	±SD	Average	±SD	ANOVA	Average	±SD	Average	±SD	Average	±SD	ANOVA
C14:0	1.56	±0.56	2.08	±0.54	1.89	±0.36	*p = 0.47*	1.48	±0.17	1.68	±0.20	1.63	±0.16	*p = 0.49*
C15:0	0.20	±0.09	0.18	±0.09	0.22	±0.10	*p = 0.85*	0.46	±0.07	0.46	±0.06	0.49	±0.05	*p = 0.85*
C16:0 (PA)	24.50	±0.54	24.06	±0.15	25.01	±0.23	*p = 0.15*	26.18a	±0.97	29.56b	±0.20	28.20ab	±0.85	*p = 0.030*
C17:0	0.70	±0.19	0.58	±0.17	0.49	±0.13	*p = 0.33*	0.57	±0.08	0.66	±0.07	0.73	±0.07	*p = 0.17*
C18:0 (SA)	14.53	±0.54	18.29	±2.10	19.20	±1.55	*p = 0.064*	16.24a	±0.85	17.26ab	±0.36	18.44b	±0.49	*p = 0.017*
C20:0	0.56	±0.14	0.35	±0.04	0.48	±0.01	*p = 0.20*	0.55	±0.11	0.42	±0.02	0.37	±0.00	*p = 0.061*
C24:0	3.92	±0.57	2.78	±0.19	2.52	±0.30	*p = 0.070*	0.78a	±0.15	0.42b	±0.04	0.45b	±0.02	*p = 0.017*
Total SFA	46.17a	±0.21	47.41b	±0.10	49.14c	±0.31	p = 0.0021	*46.27*	±2.10	50.81	±0.15	50.04	±1.36	*p = 0.062*
C16:1ω-9	8.00a	±0.06	5.41b	±0.89	5.56b	±0.71	*p = 0.022*	7.33	±0.38	4.22	±0.26	4.53	±0.40	*p = 0.0004*
C18:1ω-9 (OA)	28.14a	±0.36	21.62b	±1.57	23.72ab	±1.39	*p = 0.028*	23.56a	±0.46	15.99b	±0.80	18.84c	±0.63	*p = 0.0002*
C18:1ω-7	6.26a	±0.20	4.95b	±0.08	4.61c	±0.04	*p < 0.0001*	6.93a	±0.40	5.33b	±0.19	5.21b	±0.14	*p = 0.0010*
C24:1ω-9	1.40	±0.04	1.18	±0.32	1.01	±0.30	*p = 0.24*	1.23	±0.17	1.22	±0.28	1.08	±0.15	*p = 0.69*
Total MUFA	43.88a	±0.20	33.46b	±2.04	35.32b	±2.02	p = 0.016	39.05a	±1.41	26.76b	±1.03	29.67b	±1.29	*p = 0.0003*
C18:2ω-6 (LA)	0.33a	±0.08	0.24a	±0.02	1.08b	±0.02	*p = 0.0007*	1.00a	±0.26	0.91a	±0.10	2.17b	±0.27	*p = 0.0016*
C20:2ω-6	3.99a	±0.09	2.39b	±0.03	2.44b	±0.02	*p = 0.0001*	1.27a	±0.27	0.42b	±0.14	0.42b	±0.07	*p = 0.0028*
C20:3ω-6 (DGLA)	1.02	±0.07	1.34	±0.13	1.31	±0.06	*p = 0.059*	1.97ab	±0.20	1.77a	±0.11	2.13b	±0.04	*p = 0.035*
C20:4ω-6 (AA)	1.01a	±0.02	6.44b	±0.44	1.70a	±0.87	*p = 0.0020*	3.96a	±0.18	8.65b	±0.28	3.76a	±0.35	*p < 0.0001*
C22:4ω-6 (ADA)	0.49a	±0.02	6.64b	±0.92	0.77a	±0.31	*p = 0.0001*	1.42a	±0.14	6.71b	±0.22	1.22a	±0.18	*p < 0.0001*
C22:5ω-6 (ω-6 DPA)	0.34a	±0.23	1.03b	±0.28	0.45a	±0.14	*p = 0.019*	0.54a	±0.29	1.32b	±0.07	0.47a	±0.15	*p = 0.0032*
Total ω-6	7.04a	±0.21	17.26b	±1.32	7.02a	±0.50	p = 0.0017	*10.18a*	±0.24	19.77b	±0.62	10.17a	±0.45	*p < 0.0001*
C18:3ω-3 (ALA)	2.01a	±0.11	1.23b	±0.17	1.13b	±0.10	*p = 0.0003*	0.98	±0.21	0.89	±0.03	0.93	±0.17	*p = 0.81*
C20:4ω-3	0.19	±0.09	0.13	±0.01	0.10	±0.003	*p = 0.29*	0.38a	±0.09	0.18b	±0.03	0.20ab	±0.08	*p = 0.045*
C20:5ω-3 (EPA)	0.14a	±0.00	0.11a	±0.06	0.41b	±0.06	*p = 0.0026*	0.26a	±0.14	0.16a	±0.06	0.62b	±0.09	*p = 0.0044*
C22:5ω-3 (ω-3 DPA)	0.34a	±0.02	0.84b	±0.05	0.90b	±0.11	*p = 0.0036*	1.08	±0.14	0.97	±0.10	1.22	±0.11	*p = 0.095*
C22:6ω-3 (DHA)	0.33a	±0.18	0.27a	±0.07	6.01b	±0.68	*p < 0.0001*	1.81a	±0.15	1.52a	±0.29	6.88b	±0.73	*p < 0.0001*
Total ω-3	2.85a	±0.10	2.53a	±0.13	8.53b	±0.86	p < 0.0001	4.51a	±0.45	3.71a	±0.42	9.85b	±0.64	*p < 0.0001*
Total PUFA	9.84a	±0.25	19.80b	±1.19	15.54b	±1.71	*p = 0.0086*	14.69a	±0.69	23.48b	±1.01	20.02c	±1.08	*p = 0.0006*
C22:6ω-3:C20:4ω-6	0.23a	±0.09	0.04a	±0.01	4.92b	±0.06	*p < 0.0001*	0.46a	±0.02	0.16b	±0.00	1.76c	±0.03	*p < 0.0001*
ω-3 PUFA:ω-6 PUFA	0.40a	±0.01	0.15b	±0.02	1.21c	±0.09	p = 0.0005	0.44a	±0.03	0.18b	±0.00	0.97c	±0.02	*p < 0.0001*

Different letters indicate that groups are significantly different. Differences were assessed for significance using one-way analysis of variance followed by post-hoc Tukey's test. *p*-value of <0.05 was considered statistically significant.
